# Starch-concentration-controlled growth of anisotropic bismuth nanoplates: a facile and bio-assisted aqueous route to morphology-tuned optical response

**DOI:** 10.1039/d6ra08893g

**Published:** 2026-10-29

**Authors:** Md. Mahiuddin, Prianka Saha, Bungo Ochiai

**Affiliations:** a Chemistry Discipline, Khulna University Khulna-9208 Bangladesh mahiuddin@chem.ku.ac.bd; b Department of Applied Chemistry, Chemical Engineering, and Biochemical Engineering, Graduate School of Science and Engineering, Yamagata University Yonezawa Yamagata 992-8510 Japan ochiai@yz.yamagata-u.ac.jp

## Abstract

Herein, we report a bio-assisted aqueous synthesis of single-crystalline, anisotropic bismuth (Bi^0^) nanoplates using starch as a dual reductant/kinetic regulator and poly(vinylpyrrolidone) (PVP) as a facet-selective capping agent. Powder XRD confirms the formation of rhombohedral elemental bismuth (JCPDS Card No. 44-1246) with no detectable crystalline impurity phases. Systematic evaluation of synthesis parameters reveals a surface-energy-driven growth mechanism governed by a kinetic and capping interplay. Lower starch concentrations produce predominantly quasi-spherical nanoparticles (∼80 nm) alongside minor polyhedral structures, whereas higher starch feeds favor the formation of uniform hexagonal nanoplates with lateral diagonal dimensions of 145–240 nm. Concurrently, lower PVP feeds result in mixed morphologies containing irregular nanoplates (∼115 nm), whereas increasing the PVP feed promotes effective facet passivation, increasing the prevalence of uniform hexagonal nanoplates (120–145 nm). Time-dependent observations show an initial stage at 2 h consisting of a mixed morphology (∼145 nm) that progresses beyond 2 h into predominantly uniform hexagonal nanoplates (∼200 nm), exhibiting a visibly increased thickness plates at 24 h consistent with Ostwald ripening coupled with facet-selective capping. This structural transition induces a prominent bathochromic shift in the localized surface plasmon resonance (LSPR) from 410 nm to ∼600 nm, driven by 2D shape anisotropy and in-plane dipolar modes. Spectroscopic (FTIR) and thermal (TGA) characterizations confirm dual-polymer surface stabilization, where starch binds *via* hydroxyl hydrogen bonding (redshifted from 3380 cm^−1^ to 3290 cm^−1^) and PVP undergoes physical adsorption (1655 cm^−1^ C=O stretch), imparting high aqueous dispersion stability (−50 mV zeta potential). This study provides quantitative mechanistic insights into the green, shape-controlled synthesis of functional bismuth nanostructures for potential optoelectronic, thermoelectric, and photothermal applications.

## Abstract 2

Herein, we report a bio-assisted aqueous synthesis of single-crystalline, anisotropic bismuth (Bi^0^) nanoplates using starch as a dual reductant/kinetic regulator and poly(vinylpyrrolidone) (PVP) as a facet-selective capping agent.

## Graphical abstract

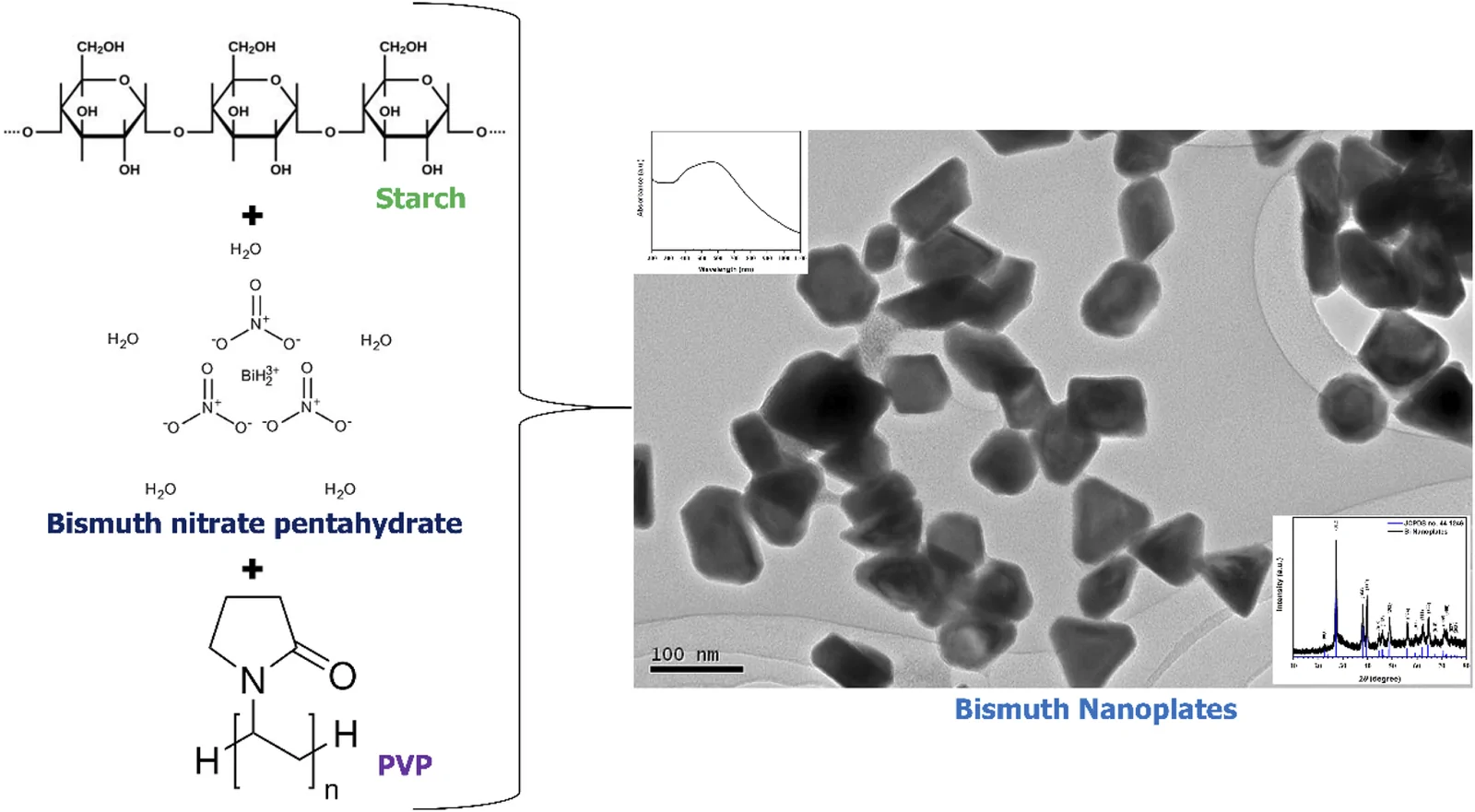

## Introduction

The synthetic strategies and outstanding properties of nanocrystalline materials have drawn considerable attention in recent decades. The characteristics of nanocrystals differ from their bulk analogs, and their prospective uses in information storage, electronics, photography, catalysis, and biological labeling have all stoked interest in them.^1–6^ Many synthetic approaches have been investigated for transition metals such as Ag, Au, Pt, and Pd, and their alloys.^7–13^ However, less research has been conducted on the synthesis of main group metal nanoparticles. The heaviest element in the main group is bismuth (Bi). In bulk, Bi has an anisotropic effective electron mass and a small band overlap, exhibiting semimetallic behavior. Furthermore, because of quantum confinement, Bi crystals transform from semimetal to semiconductor as they become smaller in size.^4,14–19^ Numerous methods have been devised for the synthesis of Bi nanostructures; among them, the solution-phase chemical reduction approach is promising for producing bulk quantities of Bi nanostructures with a variety of well-defined morphologies.^2,6,14,17,18,20–22^ Nevertheless, there has been a lack of development in the solution-phase chemical reduction pathways used to synthesize nanostructured Bi. Some research groups have recently fabricated bismuth nanoparticles using organic solvents and bismuth salts in a solution-phase chemical reduction process.^2,4,14,19,23–28^ Aqueous-phase chemical reduction is more desirable to avoid the use of organic solvents. Even for aqueous synthesis, toxic reducing agents such as sodium hypophosphite, hydrazine, and sodium borohydride have been utilized for the formation of various nanostructured Bi compounds in the majority of these methods.^20,29–36^

To address this issue, plant sources, microorganisms, and safe chemicals have been employed as reductants in the aqueous-phase chemical fabrication of nanostructured Bi. Fu *et al.* synthesized triangular Bi nanoplates using ascorbic acid as a reducing agent and PVP as a stabilizer, where ethylenediaminetetraacetic acid disodium salt (Na_2_EDTA) was used as a complexing agent.^18^ Liang *et at.* employed a direct aqueous-phase chemical reduction approach to synthesize ultrasmall Bi nanodots using d-glucose as a reducing agent and PVP as a capping agent.^33^ Shakibaie *et al.* and Nazari *et al.* used microorganisms to synthesize Bi nanoparticles.^37,38^ Recently, our research group used lemon juice as a reducing and capping agent to synthesize crystalline Bi nanoparticles.^39^ However, owing to the numerous applications of Bi nanomaterials, these few reports are insufficient to comprehend or establish a simple aqueous-phase synthetic technique for the development of nanostructured Bi. Therefore, further research is needed to synthesize nanostructured Bi using more viable, simple, and facile techniques.

Inspired by the aforementioned features, we report a unique, innovative, and simple direct aqueous-phase method for single-crystalline Bi nanoplates with starch as a safe and green reducing agent and a kinetic regulator as well with the presence of PVP as an additional capping agent. Although starch has previously been employed as a green stabilizing agent for spherical noble metal nanoparticles, its capability to direct two-dimensional anisotropic growth in elemental bismuth without harsh co-reducing agents remains underexplored. In this work, we demonstrate that starch acts synergistically with PVP to regulate nucleation kinetics and facet growth, yielding morphology-controlled Bi nanoplates. A comparison of this approach with previously reported aqueous bismuth synthetic strategies is summarized in Table 1. The nanostructured Bi was characterized by X-ray diffraction (XRD), scanning electron microscopy (SEM), transmission electron microscopy (TEM), high-resolution TEM (HRTEM), selected area electron diffraction (SAED), ultraviolet-visible (UV-vis) and Fourier-transform infrared (FTIR) spectroscopy, and thermogravimetric analysis (TGA). The particle growth behavior was controlled by tuning the reaction parameters, namely, reaction time, capping agent concentration, and reductant concentration.

**Table 1:** Comparison of aqueous bismuth nanostructure synthetic routes

Synthetic route	Reducing; capping/complexing agents	Reaction temp. & pH	Morphology	Key limitations/features	Ref.
Hydrazine reduction	N_2_H_4_, PVP, citric acid	25 °C, acidic	Spherical/Irregular	Uses toxic hydrazine reagent	40
Sodium borohydride reduction	NaBH_4_, dextran, glycine	25 °C, pH ∼ 9.0	Spheres, triangles, hexagons, and rods	Uses toxic borohydride reagent	41
	NaBH_4_, starch	25 °C, pH ∼ 9.0	Irregular shape	Aggregation, needs dialysis	42
Polyol synthesis	EG, NaBH_4_, PVP	190 °C, basic	Nanowires	High energy input; strong reductant	43
Electrodeposition	Tartaric acid, citric acid and EDTA-2Na		Dendritic with spindle	Costly, difficult for large scale production	44
	—		Hexagons		6
Hypophosphite reduction	H_2_C_4_H_4_O_6_, NaH_2_PO_2_·H_2_O	60 °C, basic	Irregular	Uses toxic hypophosphite reagent	45
	H_2_C_4_H_4_O_6_, NaH_2_PO_2_·H_2_O	60 °C, pH ∼ 2.5	Rod shaped	Uses toxic hypophosphite reagent	36
Safe chemical-based reduction	Ascorbic acid, EDTA-2Na, PVP	100 °C, pH ∼ 10–11	Triangular nanoplates	Use of multiple synthetic additives	18
	d-Glucose	70 °C, basic	Nanodots	Low yield	33
**Starch reduction**	**Starch/PVP**	**100 °C, pH** ∼ **13**	**Anisotropic nanoplates**	**Toxic-reductant-free; tunable LSPR**	This work

## Experimental section

### Materials

Bi(NO_3_)_3_·5H_2_O and NaOH were purchased from Kanto Chemical (Tokyo, Japan). Starch (soluble) was purchased from Fujifilm Wako Pure Chemicals (Osaka, Japan), and polyvinylpyrrolidone (PVP) (*M*_w_ = 50 000) was purchased from Sigma-Aldrich (St. Louis, MO, USA). All reagents were of analytical grade and used without further purification. In all experiments, deionized water was used.

### Synthesis of Bi nanoplates

The synthesis conditions are summarized in Table 2. In a typical procedure, Bi(NO_3_)_3_·5H_2_O (121 mg, 0.250 mmol) and PVP were sequentially added to an aqueous starch solution. Then, the volume was adjusted to 50 mL using deionized distilled water, and the pH of the mixture was adjusted to 13 using 4 M aqueous NaOH. The mixture was refluxed with constant stirring in a thermostat bath at the boiling point for 2–24 h under aerobic conditions. The resulting brownish-black reaction mixture was cooled to room temperature with constant stirring and centrifuged to precipitate the product from the suspension. The precipitate was washed four times with water. The product was vacuum-dried for 12 h at 60 °C. Control experiments were conducted in the absence of either starch or PVP.

**Table 2:** Consolidated experimental parameters for bismuth nanostructure synthesis

Product type	Bi(NO_3_)_3_·5H_2_O	Starch content	PVP content	NaOH conc	Reaction time	Stirring speed/temp	Centrifugation/wash protocol
Time-dependent	121 mg (0.250 mmol)	1.5 g	1.0 g	4.0 M (pH ∼ 13)	2 h	300 rpm, 100 °C	12 000 rpm/15 min; 4× H_2_O
					4 h		
					8 h		
					16 h		
					24 h		
Starch content-dependent	121 mg (0.250 mmol)	1.0 g	1.0 g	4.0 M (pH ∼ 13)	2 h	300 rpm, 100 °C	12 000 rpm/15 min; 4× H_2_O
		1.5 g					
		2.0 g					
		2.5 g					
PVP content-dependent	121 mg (0.250 mmol)	1.5 g	0.3 g	4.0 M (pH ∼ 13)	2 h	300 rpm, 100 °C	12 000 rpm/15 min; 4× H_2_O
			0.6 g				
			1.0 g				
			1.7 g				

### Measurements

XRD analysis was conducted on a Rigaku (Tokyo, Japan) MiniFlex 600 diffractometer with Cu-Kα radiation using powder of resultant product. SEM measurements were conducted on a Hitachi (Tokyo, Japan) SU-8000 scanning electron microscope at accelerating voltages of 10 and 20 kV. For SEM sample preparation, the dried products were re-dispersed in water at the same product-to-water ratio for all samples. The resulting dispersions were drop-cast onto aluminum sheets, vacuum-dried, and then coated with gold. Energy-dispersive X-ray spectroscopy (EDX) analysis was conducted on a JEOL (Tokyo, Japan) JSM-6510A analytical scanning electron microscope equipped with a JEOL JED2300 EDX spectrometer operated at an acceleration voltage of 10 kV using a compressed disk of resultant dry product as a sample placed on an SEM pin with the help of double-sided carbon tape. To eliminate background carbon interference during quantification, the dry product was analyzed in the form of a compressed pellet (>10 µm thickness). The sample thickness ensured that the electron beam excitation volume remained entirely within the specimen, avoiding signal collection from the underlying mounting carbon substrate. TEM measurements were conducted on a JEOL TEM-2100F field-emission electron microscope. TGA was carried out on a Seiko Instruments (Tokyo, Japan) TG/DTA 6200 (EXSTER6000) thermogravimetric analyzer at a heating rate of 10 °C min^−1^ under N_2_. Zeta potentials were measured by dynamic light scattering (DLS) analysis conducted on a Malvern (Malvern, UK) Zetasizer Nano ZS instrument. UV-vis spectroscopic analyses were carried out on a HACH DR 5000 (Loveland, CO, USA) UV-visible spectrometer. Absorption spectra were recorded at a resolution of 1 nm within 200–1100 nm. Fourier-transform infrared (FTIR) spectra were recorded on a JASCO (Tokyo, Japan) FT/IR-460 plus spectrometer using a KBr pellet with a scan rate of 4 cm^−1^ s^−1^ at 25 °C. Centrifugation was performed on a KUBOTA (Osaka, Japan) 3700 microrefrigerated centrifuge machine.

## Results and discussion

### Structural and morphological analyses of Bi nanoplates

Treatment of Bi(NO_3_)_3_·5H_2_O with starch in the presence of PVP yielded a black product. A black product also which was not obtained in the absence of starch. The powder XRD pattern (Fig. 1) matches rhombohedral elemental bismuth (Bi^0^, JCPDS Card No. 44-1246, space group *R*3̄*m*).^25,46^ No detectable crystalline impurity phases (such as Bi_2_O_3_) were observed within the resolution limits of powder XRD. The XRD study revealed that soluble starch effectively reduced Bi^3+^ to form nanostructured Bi, which was confirmed as Bi nanoplates, as described later. While XRD confirms bulk phase purity and zero-valent crystalline metallic identity, surface compositional features and potential trace surface oxidation were further evaluated *via* spectroscopic methods.

**Fig. 1:**
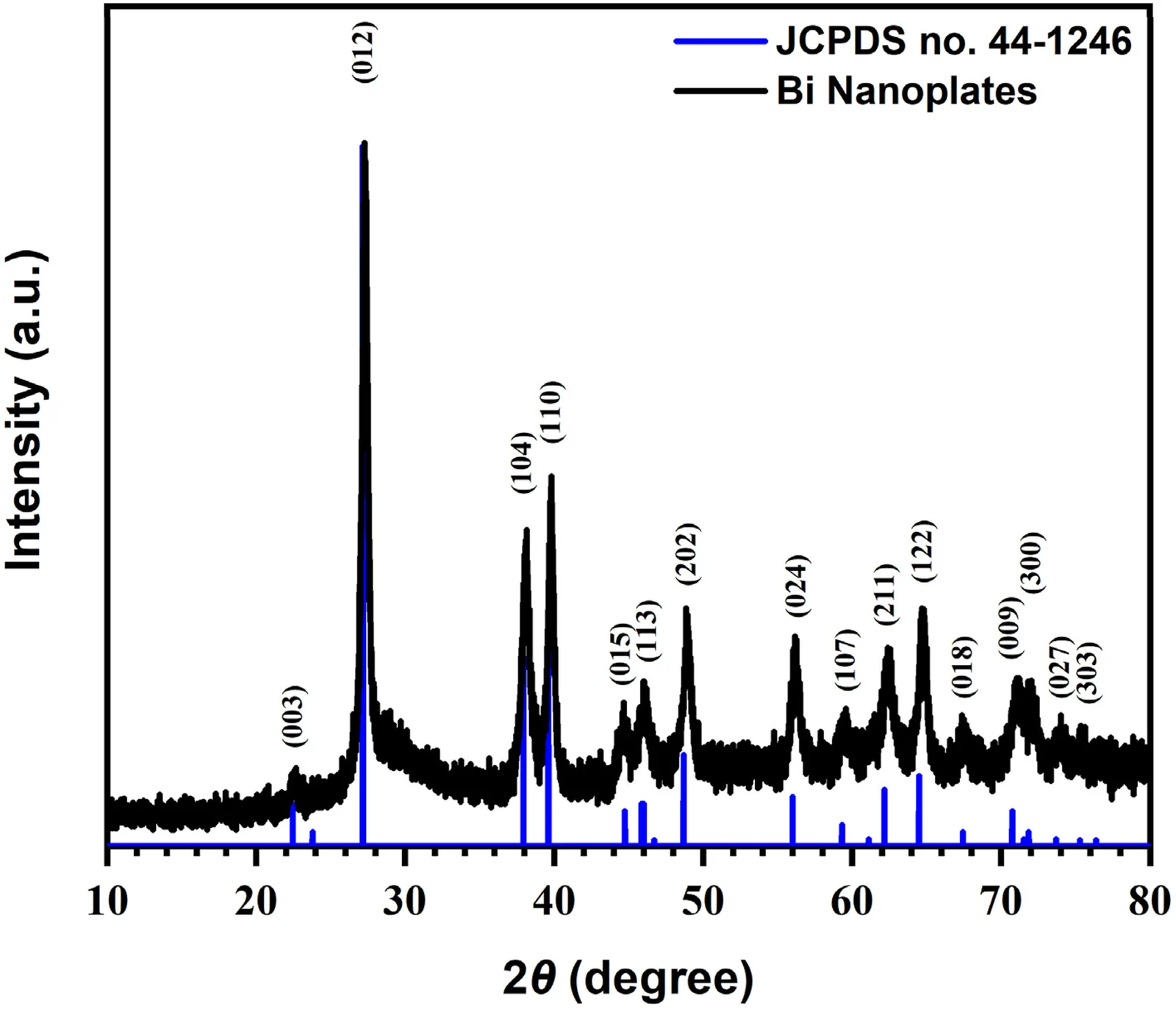
XRD pattern of the bismuth nanoplates synthesized using starch and PVP (conditions: Bi(NO_3_)_3_·5H_2_O = 0.250 mmol, starch = 1.5 g, PVP = 1.0 g, temperature = 100 °C, pH = 13, time = 2 h).

The optical properties of the nanostructured Bi were characterized by UV-vis spectroscopy. Surface plasmon resonance (SPR) bands of Bi nanomaterials depend on their size and morphologies as follows. Bi nanoparticles with a size smaller than 20 nm exhibit an SPR band around 196–300 nm, and Bi nanospheres exhibit two absorption peaks around 420 and 600 nm, varying on the size over 100 nm and peaks red-shifted as the particle size increases.^47^ On the other hand, Bi nanodisks with 70–160 nm diagonal length exhibit absorption peak at 603 nm, but theoretically, nanodisks should have two different absorption peaks.^48^ In addition, Bi nanopolyhedra having the size range of 120–250 nm exhibit two absorption peak at 451 nm and 649 nm.^2,29^ The UV-vis spectrum of the obtained nanostructured Bi (Fig. 2) dispersed in water shows a broad peak with a peak top at 590 nm and a shoulder at 412 nm, agreeing with the presence of Bi nanomaterials having the size larger than 100 nm along with smaller particles.

**Fig. 2:**
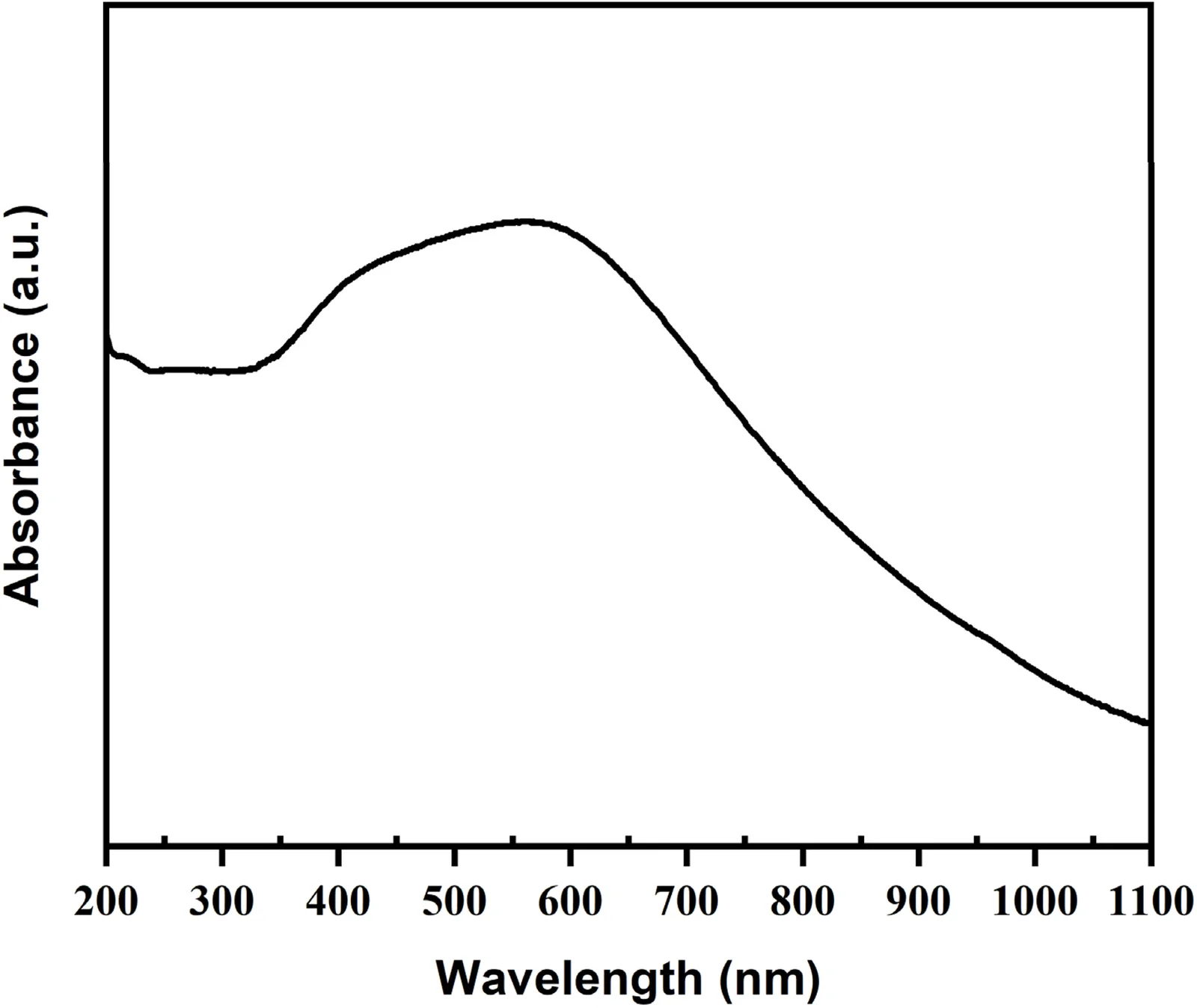
UV-vis spectrum of aqueous dispersion of the bismuth nanoplates synthesized using starch and PVP (conditions: Bi(NO_3_)_3_·5H_2_O = 0.250 mmol, starch = 1.5 g, PVP = 1.0 g, temperature = 100 °C, pH = 13, time = 2 h).

The dimensions and morphology of the obtained nanostructured Bi were analyzed by electron microscopy. The representative SEM image of the Bi nanostructures synthesized using starch and PVP (Fig. 3b) reveals faceted polygonal nanoplates with lateral diagonal lengths spanning 80–150 nm. Among these geometries, hexagonal nanoplates constitute the predominant morphology, displaying a mean lateral diagonal dimension of ∼145 nm (Fig. S1a). In contrast, the product obtained without PVP (Fig. 3a) consisted of similar nanoparticles and larger products with irregular shapes and sizes, and this difference clearly indicates the effectiveness of PVP in the formation of well-defined nanoplates. The morphological evolution relies on a synergistic capping and kinetic regulation mechanism between starch and PVP.^40^ Starch functions as the primary reducing agent and kinetic regulator, where higher starch levels accelerate nucleation and favor anisotropic growth while PVP acts as a facet-selective capping agent that direct 2D lateral expansion into uniform hexagonal nanoplates.^2,18^

**Fig. 3:**
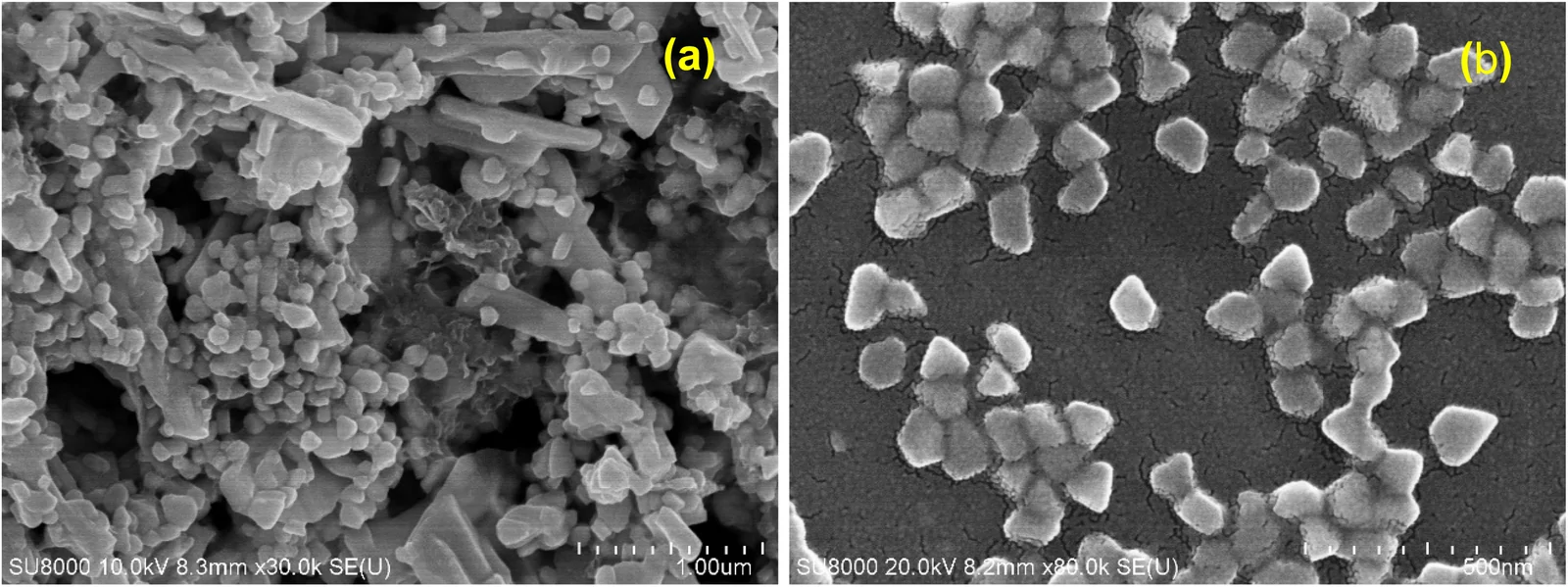
SEM images of the bismuth nanostructures synthesized using starch and PVP (a) in absence of PVP (scale bar = 1 µm) and (b) in presence of PVP (scale bar = 500 nm) (conditions: Bi(NO_3_)_3_·5H_2_O = 0.250 mmol, starch = 1.5 g, PVP = 1.0 g, temperature = 100 °C, pH = 13, time = 2 h).

The TEM image of the nanostructured Bi synthesized using starch and PVP (Fig. 4a) also shows hexagonal Bi nanoplates along with other polygonal nanoplates. The diagonal lengths of the nanoplates calculated from the TEM images were consistent with those obtained from the SEM analysis. The HRTEM image of a single hexagonal Bi nanoplate (Fig. 4b) exhibits the clear lattice fringes of 0.313 nm, complying well with the *d*-spacing of the (012) plane of the rhombohedral elemental Bi. SAED performed on an individual representative hexagonal nanoplate exhibits a clear dot array pattern, confirming its single-crystalline nature. However, the bulk product presumably anticipated as highly crystalline anisotropic nanostructures with a high proportion of faceted nanoplates.

**Fig. 4:**
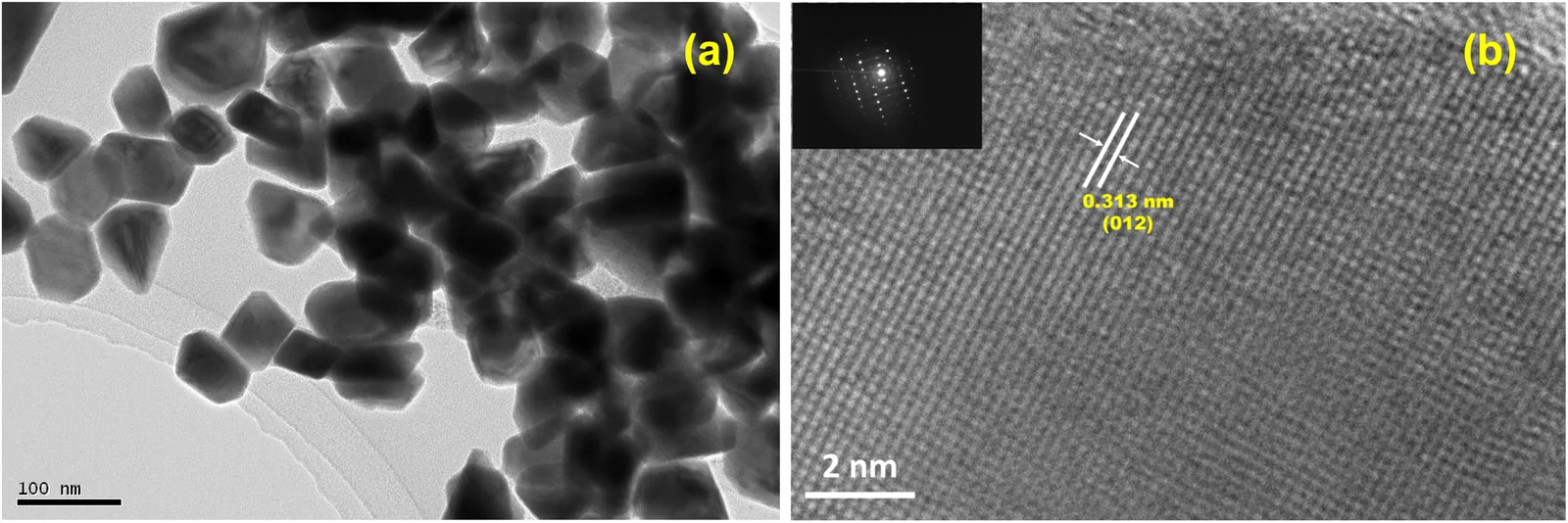
(a) TEM image of bismuth nanoplates synthesized using starch and PVP and (b) HRTEM image of a single hexagonal Bi nanoplate with its SAED pattern (conditions: Bi(NO_3_)_3_·5H_2_O = 0.250 mmol, starch = 1.5 g, PVP = 1.0 g, temperature = 100 °C, pH = 13, time = 2 h).

FTIR spectroscopic analysis of bismuth nanoplates synthesized using starch and PVP revealed the adsorption of both organic substances, as evidenced by the identical absorption bands observed in the starch and PVP spectra (Fig. 5). The starch spectrum exhibits characteristic features, including broad O–H (3300–3500 cm^−1^), C–H (2800–3000 cm^−1^), and intense C–O (1018 cm^−1^) stretching vibration bands, while the PVP spectrum displays distinctive features, including broader C–H (2800–3000 cm^−1^), sharp C=O (1651 cm^−1^), and N–C (1200–1300 cm^−1^) stretching vibration bands. The bismuth nanoplate spectrum exhibited complex patterns derived from both polymers with substantial modifications. The characteristic C=O stretching mode of PVP appears at ∼1655 cm^−1^, exhibiting a negligible shift (<1 cm^−1^) relative to pure PVP (∼1660 cm^−1^), which indicates that PVP interacts with the Bi surface primarily *via* weak physical adsorption. The low intensity of the N–C vibration (1250–1350 cm^−1^) likely stems from partial spectral overlap with the bending mode of adsorbed ambient water. In contrast, the broad hydroxyl (–OH) absorption band of starch exhibits a distinct redshift from ∼3380 cm^−1^ down to ∼3290 cm^−1^ (spanning the 2900–3500 cm^−1^ region), confirming hydrogen bonding between the hydroxyl groups and the bismuth surface. Furthermore, characteristic aliphatic C–H stretching absorption bands at 2921, 2886, and 2844 cm^−1^ confirm the dual presence of both polymeric capping agents. Critical evidence of successful synthesis appears in the non-superimposable nature of the spectrum compared to those of pure components, weak features in the 400–850 cm^−1^ region are assigned to skeletal organic deformation modes and minor surface Bi–O vibrations, and modified intensities in the fingerprint region (500–1500 cm^−1^).^18,49^ These spectral characteristics confirm that PVP and starch collectively form an effective dual capping system that not only prevents aggregation through distinct chemical bonding mechanisms but also directs the formation of the plate-like morphology through controlled growth mediation, ultimately yielding stabilized bismuth nanoplates with well-defined structural characteristics. The good colloidal stability of nanostructured Bi was also confirmed from the negative zeta potential values of −50 mV. This highly negative value implies that high electrostatic dispersion stability imparted by the surface-adsorbed organic layer, presumably the presence of carboxylate (COO^−^) groups produced by the oxidation of starch under the basic conditions. A peak around 1600 cm^−1^, unobservable in both spectra, is assignable to the carbonyl group of the carboxylate group, confirming that starch acts as the main capping agent. Moreover, the broad IR absorption around 2500–3000 cm^−1^ might originate from residual COO–H, which also supports the presence of oxidized starch on the surface of nanostructured Bi.

**Fig. 5:**
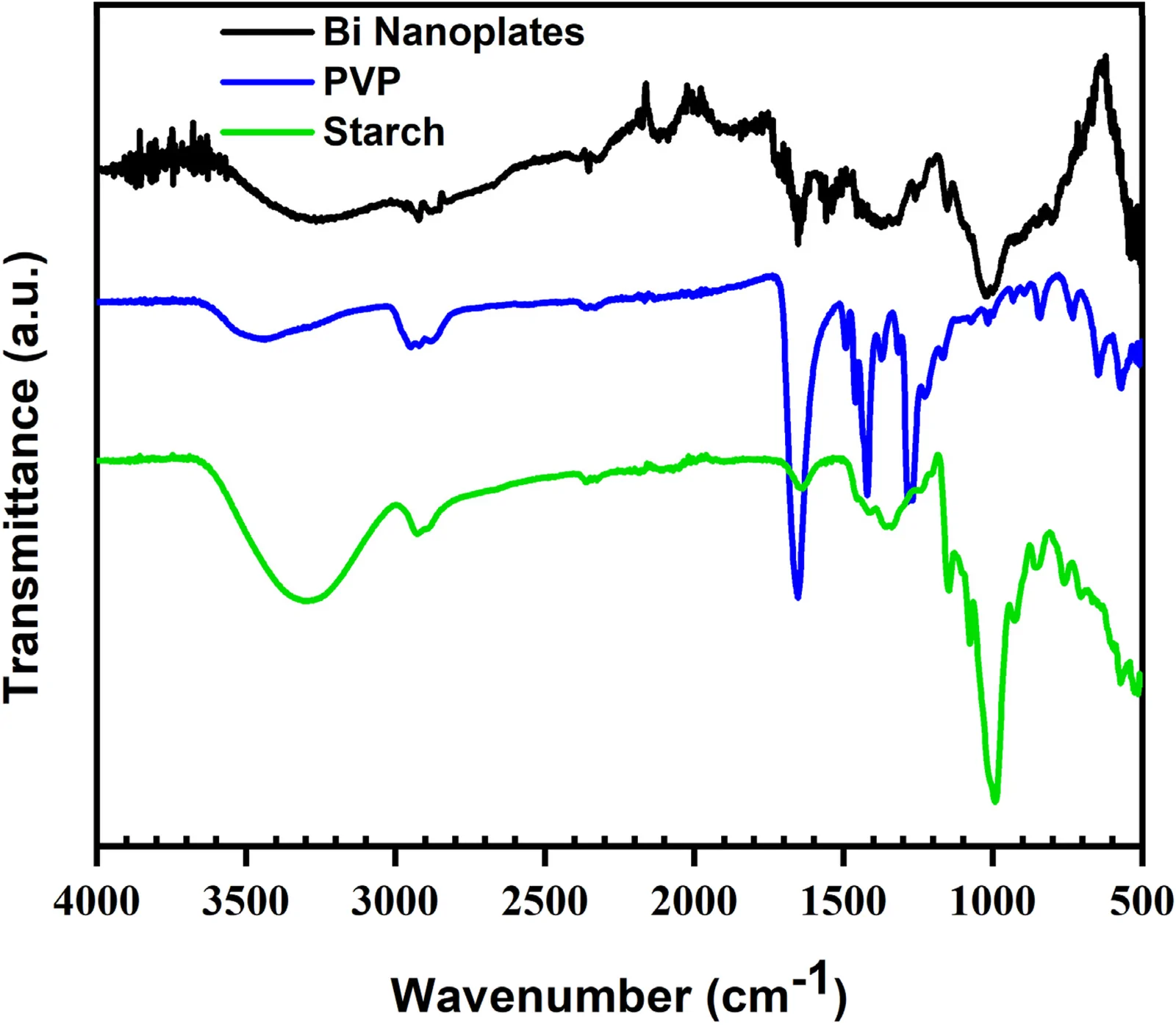
FTIR spectra of starch, PVP, and the bismuth nanoplates synthesized using starch and PVP (conditions: Bi(NO_3_)_3_·5H_2_O = 0.250 mmol, starch = 1.5 g, PVP = 1.0 g, temperature = 100 °C, pH = 13, time = 2 h).

As established in the literature, biomolecules containing diol and carboxyl functional groups promote preferential crystal growth along the (012) facet of bismuth.^40^ Starch, a polyol components and its oxidized derivatives bearing carboxyl groups, could be effectively coordinate with the metallic bismuth surface during synthesis. Consequently, the formation of two-dimensional bismuth nanoplates in this study is driven by preferential growth along the (012) orientation, a mechanism fully corroborated by our XRD and HRTEM analyses.

The presence of Bi was also confirmed by the EDX spectrum, which shows a strong Bi signal (Fig. 6). The C signal in the EDX spectrum are attributable to the presence of both starch and PVP on the surface of the Bi nanostructures, whereas O signals stem from a combination of surface-bound organic capping agents and unavoidable minor ambient surface oxidation.

**Fig. 6:**
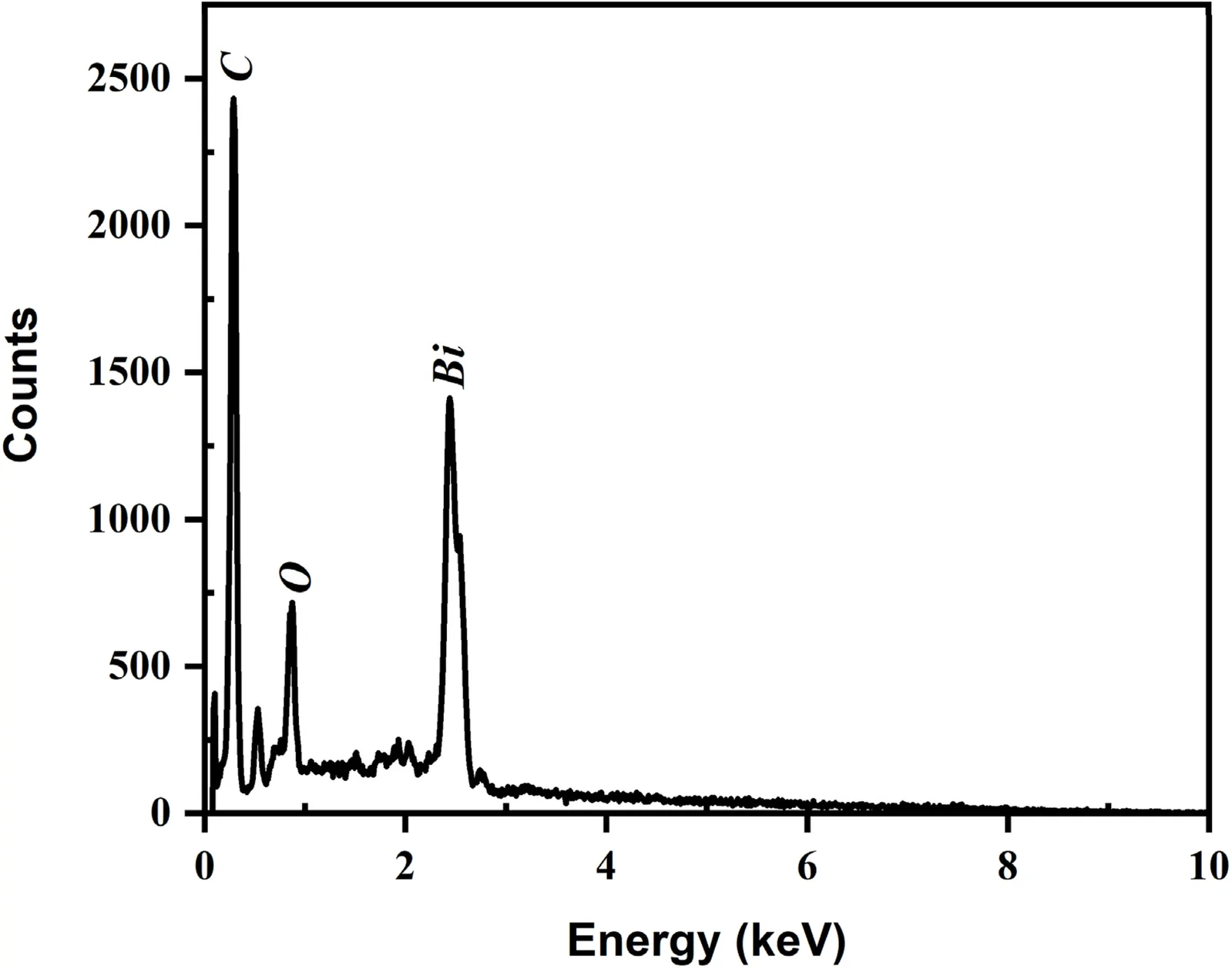
EDX spectrum of the bismuth nanoplates synthesized using starch and PVP (conditions: Bi(NO_3_)_3_·5H_2_O = 0.250 mmol, starch = 1.5 g, PVP = 1.0 g, temperature = 100 °C, pH = 13, time = 2 h).

The organic content of the obtained nanostructured Bi was determined by TGA (Fig. 7). TGA curve exhibits three distinct weight-loss regimes: (i) below 120 °C (∼2.5 wt%), corresponding to the desorption of physically adsorbed surface moisture; (ii) 120–320 °C (∼4.1 wt%), corresponding to starch thermal decomposition; and (iii) 320–500 °C (∼3.8 wt%), corresponding to the degradation of the PVP polymer backbone.

**Fig. 7:**
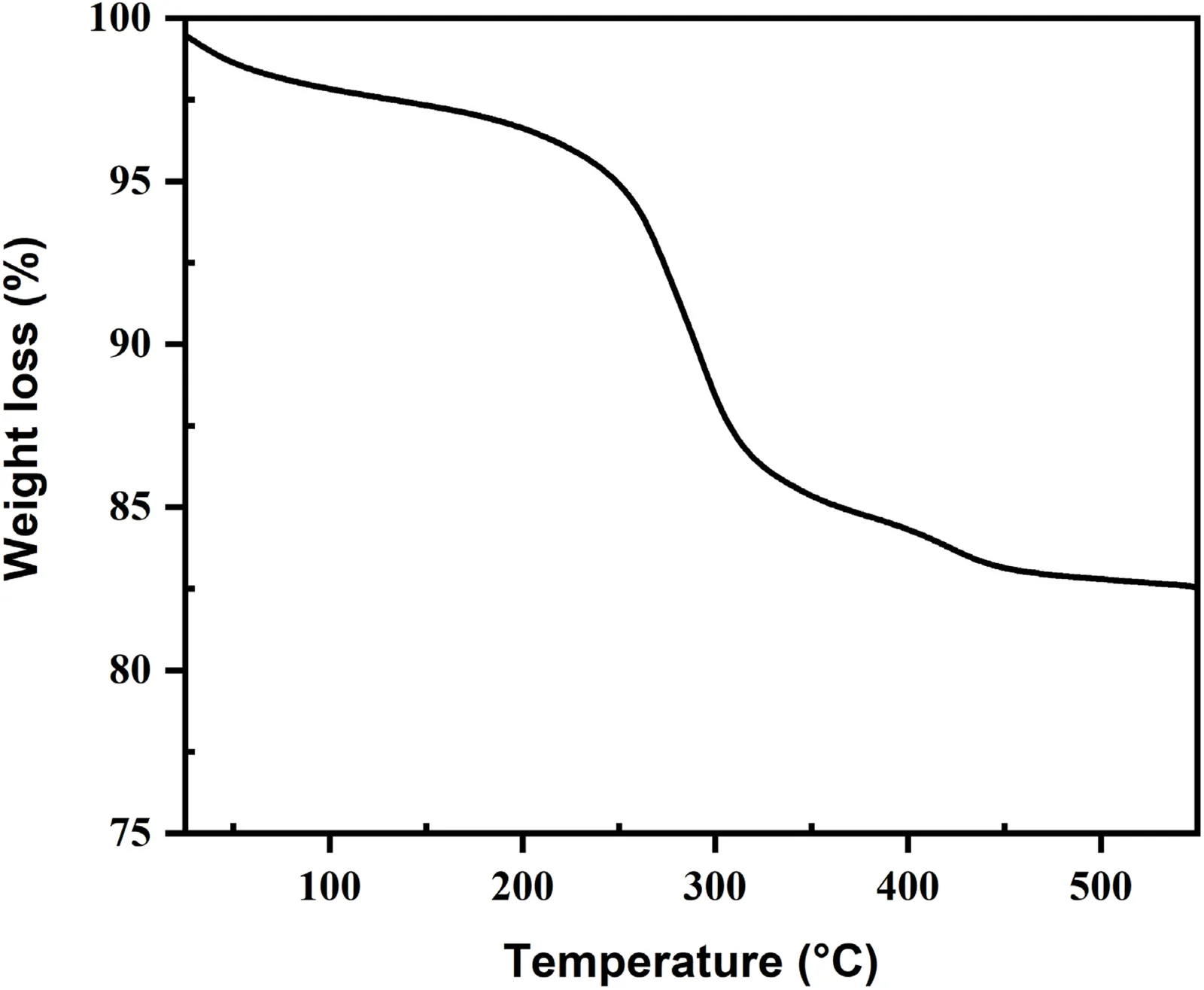
TGA curve of the bismuth nanoplates synthesized using starch and PVP (conditions: Bi(NO_3_)_3_·5H_2_O = 0.250 mmol, starch = 1.5 g, PVP = 1.0 g, temperature = 100 °C, pH = 13, time = 2 h) (10 °C min^−1^, N_2_).

### Influence of reaction time

The reaction time is an important factor in determining the size and shape of Bi products, which were synthesized under the conditions listed in Table 3.

**Table 3:** Experimental parameters for bismuth nanostructure synthesis using starch and PVP at different reaction time

Product no.	Bi(NO_3_)_3_·5H_2_O	Starch content	PVP content	NaOH conc	Reaction time
1	121 mg (0.250 mmol)	1.5 g	1.0 g	4.0 M (pH ∼ 13)	2 h
2					4 h
3					8 h
4					16 h
5					24 h

SEM analysis of the Bi products synthesized at varying reaction intervals reveals a time-dependent morphological evolution (Fig. 8). At the initial reaction stage (2 h), the synthesized Bi product exhibits a mixed morphology composed of hexagonal nanoplates alongside various polyhedral geometries (Fig. 8a), with the hexagonal nanoplates displaying an average lateral diagonal dimension of ∼145 nm (Fig. S1a). As the reaction progresses, the morphology undergoes a gradual transition into predominantly uniform hexagonal nanoplates (Fig. 8b–e). Beyond 2 h, the population fraction of hexagonal nanoplates increases significantly, settling at a larger and nearly uniform lateral diagonal dimension of ∼200 nm (Fig. S1b–e). Furthermore, the nanoplates obtained after 24 h exhibit a visibly increased thickness profile (Fig. 8e). This time-dependent structural evolution marked by lateral expansion followed by eventual vertical thickening is consistent with an Ostwald ripening process coupled with facet-selective polymer capping, where rapid initial seed formation transitions into controlled anisotropic 2D growth and eventual thermodynamic stabilization.^2,18^

**Fig. 8:**
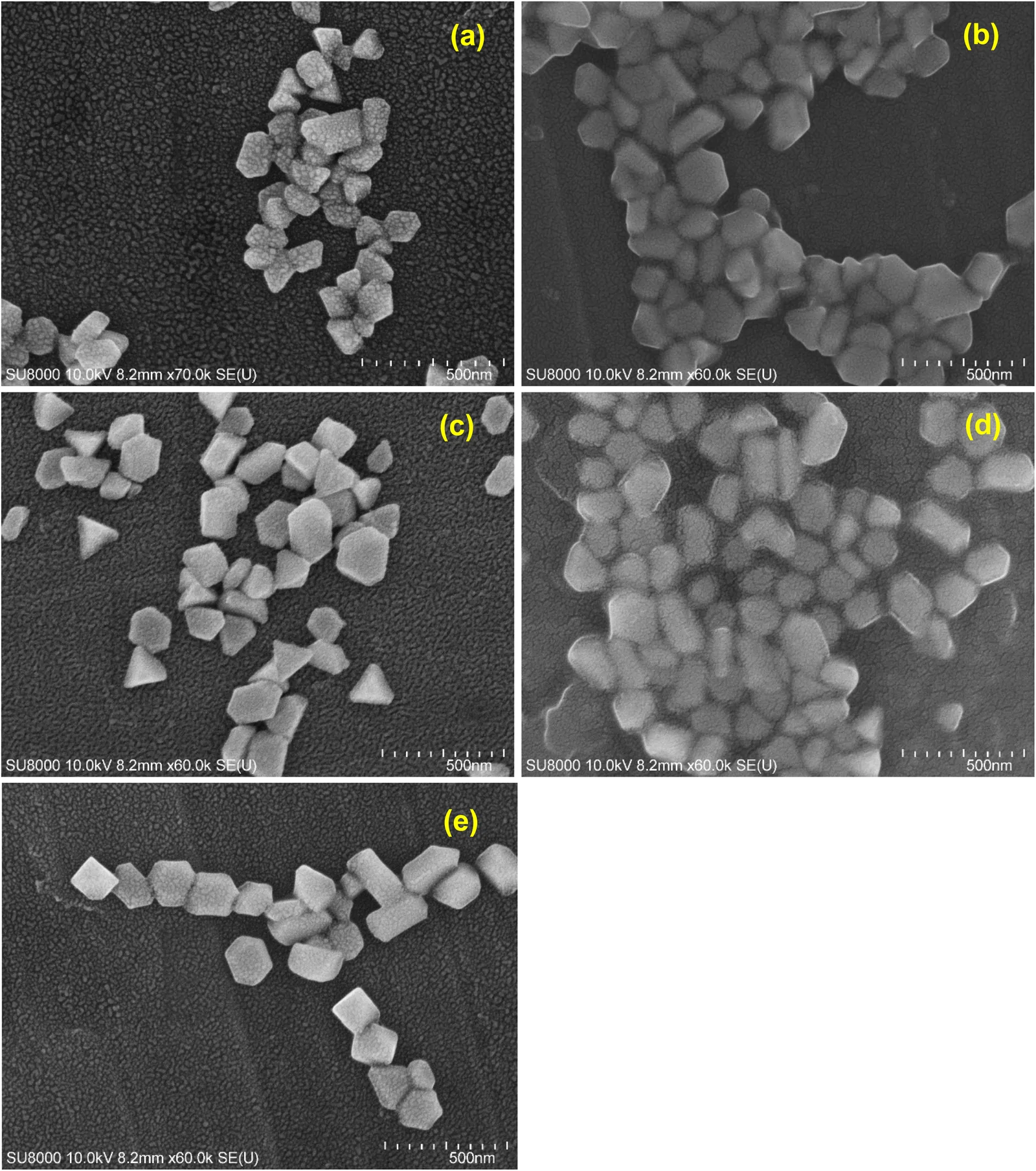
SEM images of the bismuth nanoplates synthesized using starch as a reductant in the presence of PVP at different reaction times (a) 2 h, (b) 4 h, (c) 8 h, (d) 16 h, and (e) 24 h (conditions: Bi(NO_3_)_3_·5H_2_O = 0.250 mmol, starch = 1.5 g, PVP = 1.0 g, temperature = 100 °C, pH = 13).

This morphological change correlated with the time-dependent UV-vis absorption spectra (Fig. 9). The characteristic absorption maxima of the Bi nanoplates gradually shifted toward longer wavelengths as the reaction time increased. This bathochromic shift is indicative of the change in the localized surface plasmon resonance (LSPR) properties as the crystal growth. The peak top of the product obtained at 2 h was 590 nm, and the tops of the absorption peaks shifted from 590 nm when the reaction time was extended to 4–16 h. The peak top of the product obtained at 24 h exceeded 700 nm with broadening of the peak. Furthermore, the absorption intensity increased progressively with reaction time, indicating increase in concentration of Bi nanostructures and expansion of Bi^(0)^ surface.^2^

**Fig. 9:**
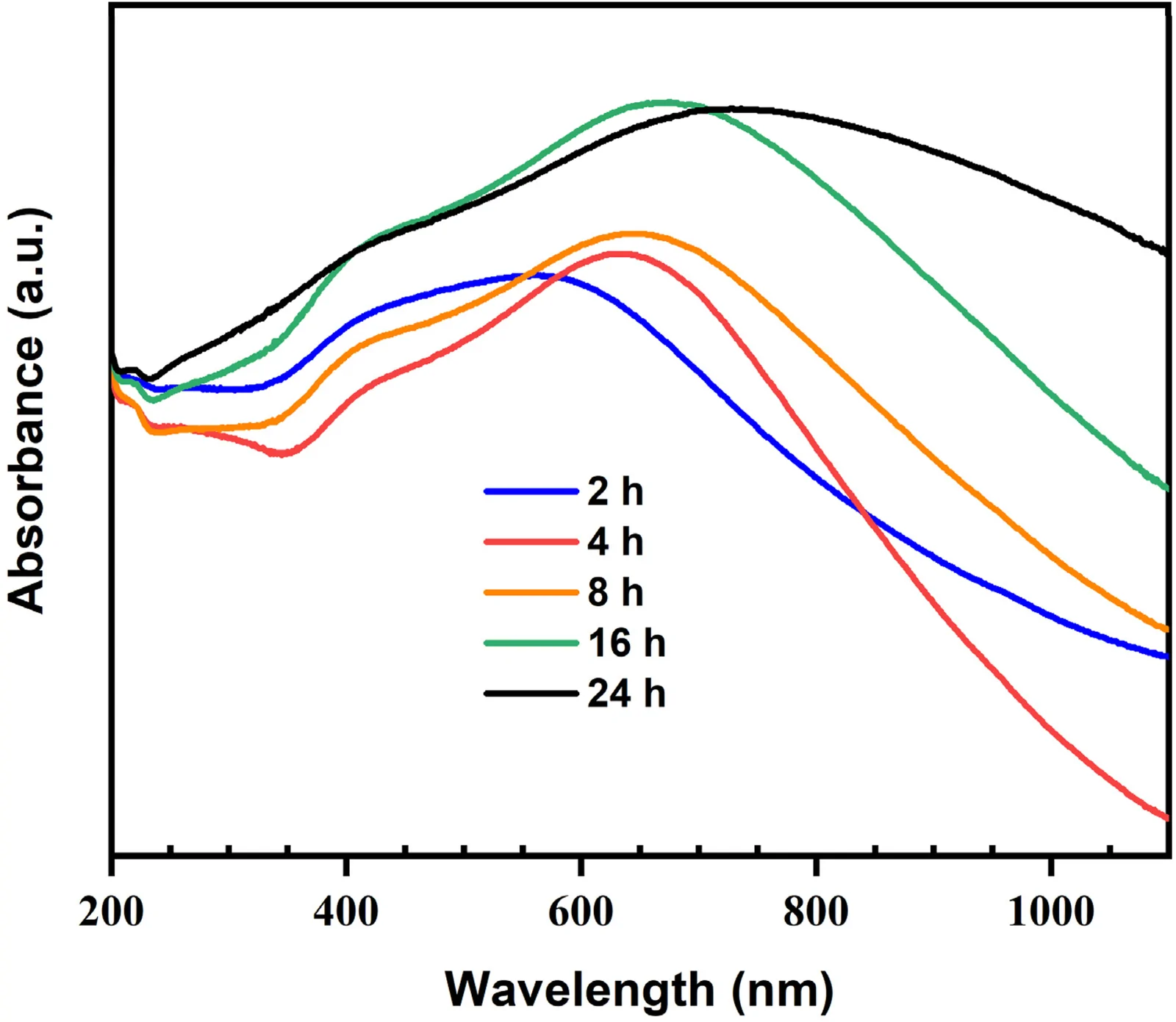
UV-vis spectra (acquired at constant concentration) of aqueous dispersions of the bismuth nanoplates synthesized using starch as a reductant in the presence of PVP at different reaction times (2–24 h) (conditions: Bi(NO_3_)_3_·5H_2_O = 0.250 mmol, starch = 1.5 g, PVP = 1.0 g, temperature = 100 °C, pH = 13).

Based on the time-dependent SEM observations and corresponding optical shifts in the UV-vis spectra, a plausible surface-energy-driven growth mechanism is proposed.^2,18,40,50^ Thermodynamically, the initial nucleus geometry is governed by the minimization of total surface free energy. Following rapid initial nucleation, the seeds undergo anisotropic, orientation-dependent growth mediated by the synergistic interplay between starch and PVP. Starch and PVP play distinct, complementary roles: starch acts as the primary reducing agent and kinetic regulator, where starch regulates the reduction kinetics and nucleation rate, whereas PVP acts as a facet-selective capping agent that preferentially adsorbs onto specific high-energy crystal faces. This selective passivating effect lowers the growth rate perpendicular to the passivated planes, thereby promoting preferential lateral expansion. Consequently, the time-dependent morphological evolution observed by SEM supports a mechanism driven by kinetic facet capping and anisotropic lateral growth along preferred crystallographic directions.^18,20^

### The influence of PVP

The size and shape of the resulting nanostructured Bi were also significantly influenced by the amount of the capping agent used, which were synthesized under the conditions listed in Table 4.

**Table 4:** Experimental parameters for bismuth nanostructure synthesis using starch with different PVP content

Product no.	Bi(NO_3_)_3_·5H_2_O	Starch content	PVP content	NaOH conc	Reaction time
1	121 mg (0.250 mmol)	1.5 g	0.3 g	4.0 M (pH ∼ 13)	2 h
2			0.6 g		
3			1.0 g		
4			1.7 g		

The SEM images of the nanostructured Bi (Fig. 10) demonstrate that lower PVP feeds result in mixed morphologies comprising irregular nanoplates alongside hexagonal geometries with an average lateral diagonal dimension of ∼115 nm (Fig. S2a). As the PVP feed is increased, uniform hexagonal nanoplates become increasingly prevalent, displaying lateral diagonal dimensions ranging from 120 to 145 nm (Fig. S2b–d). The crystallographic surfaces enclosing the particles and their forms are closely connected. Generally speaking, surface energies associated with different crystallographic planes vary. A spherical particle often exposes high-index planes, which can lead to higher surface energy; thus, faceting typically develops to maximize the exposure of low-index, lower-energy planes.^2,18,19^ PVP is known to preferentially adsorb onto specific crystal planes, directing particle growth along preferred orientations to form distinct morphologies. Presumably, at lower PVP concentrations, incomplete or inefficient molecular adsorption on Bi crystal faces leads to unpassivated growth across multiple crystal orientations, yielding mixed morphologies of quasi-spherical particles and irregular nanoplates (∼100 nm).^18,40,50^ Conversely, higher PVP concentrations ensure sufficient surface coverage to selectively passivate high-energy facets, effectively suppressing vertical growth.

**Fig. 10:**
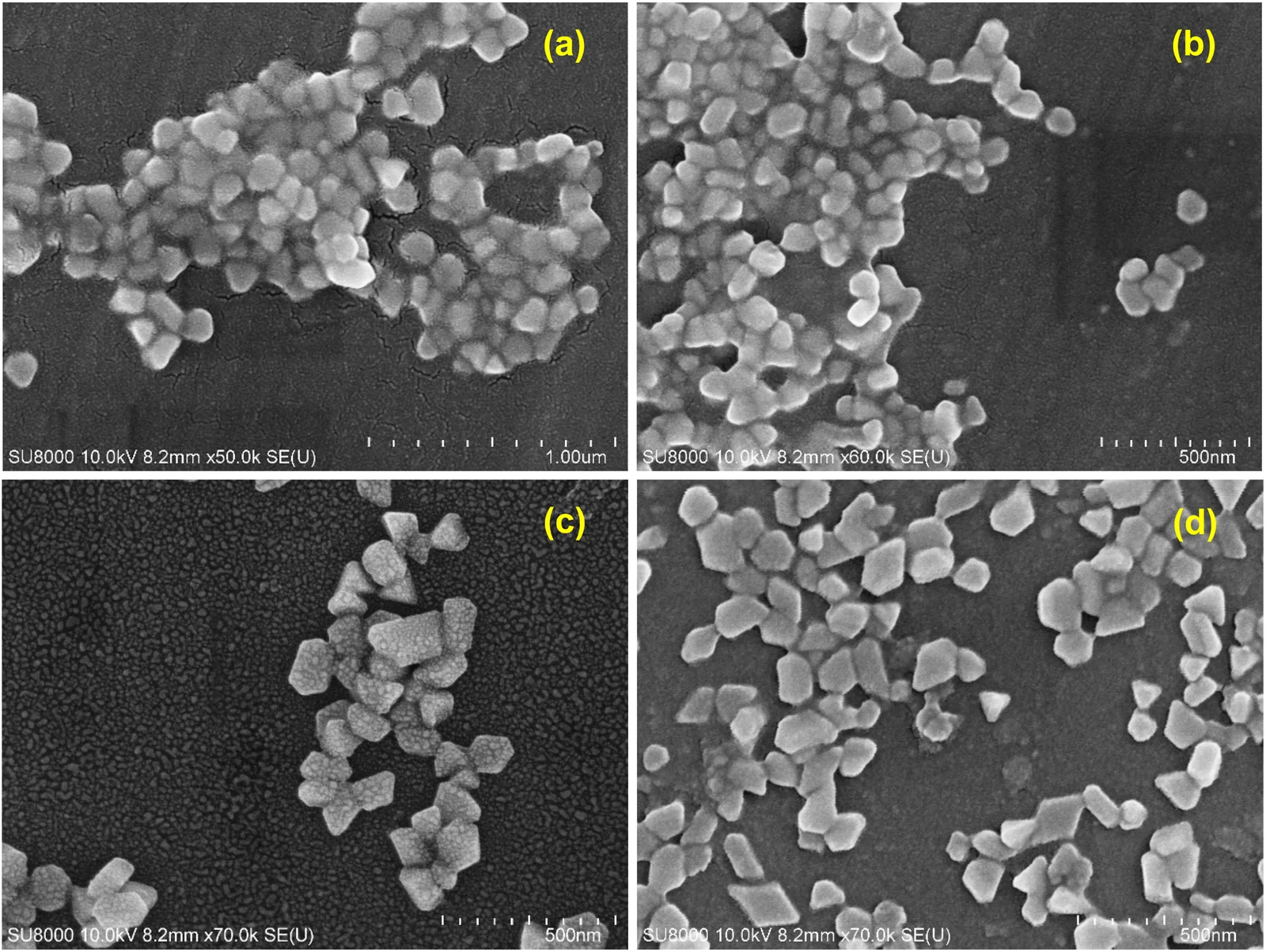
SEM images of the bismuth nanoplates synthesized using starch as a reductant in the presence of different PVP contents (a) 0.3 g, (b) 0.6 g, (c) 1.0 g, and (d) 1.7 g (conditions: Bi(NO_3_)_3_·5H_2_O = 0.250 mmol, starch = 1.5 g, time = 2 h, temperature = 100 °C, pH = 13).

The UV-vis spectra of nanostructured Bi produced with varying PVP amount (Fig. 11), in which the absorption becomes pronounced and shifts to longer wavelength as the increase of PVP feed, agrees with the morphological change confirmed by the SEM images. The lower PVP feed resulted in the weaker absorption peak at 410 nm. In contrast, higher PVP resulted in the greater absorption peak at about 600 nm. These results show that comparatively spherical particles develop when the PVP content is lower, while plate-shaped particles form when the PVP level is higher.^2^

**Fig. 11:**
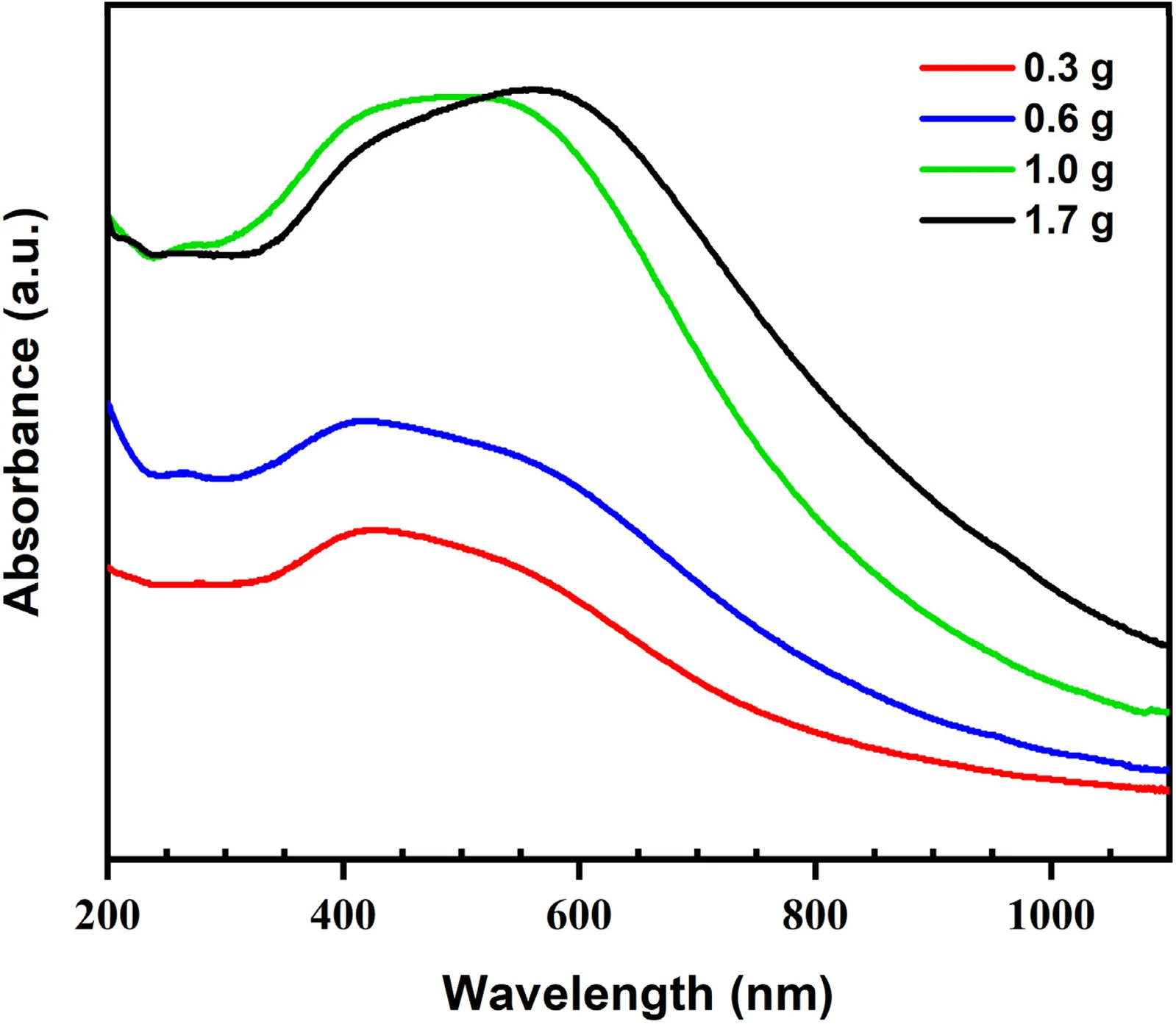
UV-vis spectra (acquired at constant concentration) of the bismuth nanoplates synthesized using starch as a reductant in the presence of different PVP contents (0.3–1.7 g) (conditions: Bi(NO_3_)_3_·5H_2_O = 0.250 mmol, starch = 1.5 g, temperature = 100 °C, pH = 13, time = 2 h).

### Influence of starch

The concentration of the reducing and stabilizing agent plays a key role in governing nucleation kinetics, particle growth, and overall morphology, which were synthesized under the conditions listed in Table 5.

**Table 5:** Experimental parameters for bismuth nanostructure synthesis using different starch content in the presence of PVP

Product no.	Bi(NO_3_)_3_·5H_2_O	Starch content	PVP content	NaOH conc	Reaction time
1	121 mg (0.250 mmol)	1.0 g	1.0 g	4.0 M (pH ∼ 13)	2 h
2		1.5 g			
3		2.0 g			
4		2.5 g			

The UV-vis spectra of the Bi nanostructures prepared with varying starch contents (Fig. 12) show distinct absorption profiles reflecting these structural variations. At lower starch concentrations, the spectrum is dominated by an absorption band near 410 nm, characteristic of smaller, quasi-spherical nanoparticles. As the starch concentration increases, a prominent bathochromic (red) shift toward approximately 600 nm is observed. This optical shift is consistent with increased particle size and shape anisotropy,^2^ supporting the formation of anisotropic, plate-like nanostructures at higher starch levels.

**Fig. 12:**
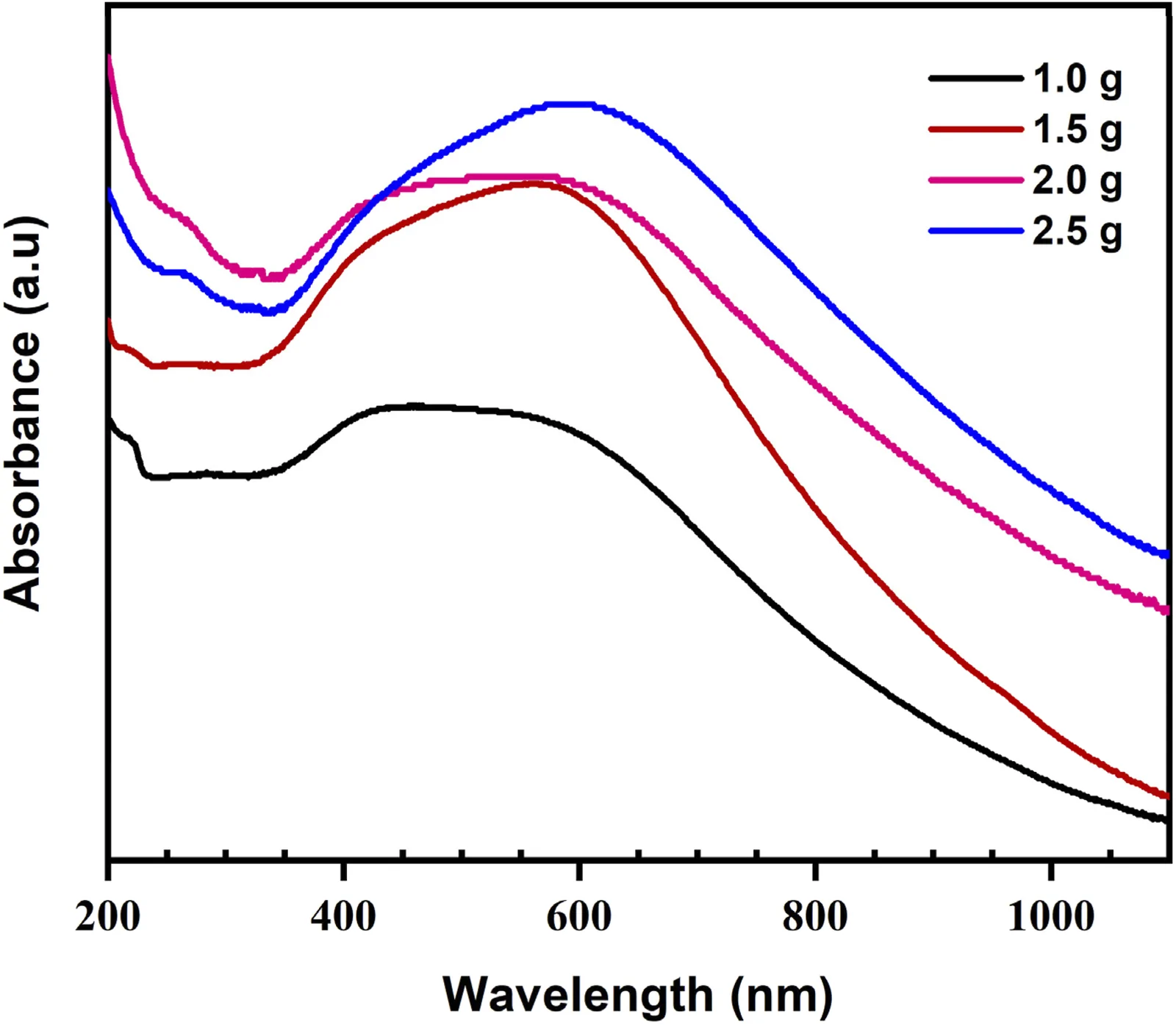
UV-vis spectra (acquired at constant concentration) of the bismuth nanoplates synthesized in the presence of PVP using different amounts of starch as a reductant (1.0–2.5 g) (conditions: Bi(NO_3_)_3_·5H_2_O = 0.250 mmol, PVP = 1.0 g, temperature = 100 °C, pH = 13, time = 2 h).

The UV-vis spectroscopy results align closely with the SEM observations. SEM images of the Bi nanostructures synthesized with varying starch contents (Fig. 13) confirm that starch concentration significantly dictates particle morphology. At lower starch concentrations, the product predominantly consists of quasi-spherical nanoparticles alongside minor polyhedral structures, with the quasi-spherical particles averaging ∼80 nm in diameter (Fig. S3a). Conversely, higher starch concentrations favor the preferential formation of uniform, hexagonal nanoplates with lateral diagonal dimensions ranging from 145 to 240 nm (Fig. S3b–d). Presumably, higher starch concentrations accelerate nucleation and facilitate rapid precursor consumption. Higher starch concentrations provide sufficient kinetic driving force for facet-controlled growth, allowing PVP to direct 2D lateral expansion into uniform hexagonal nanoplates. Conversely, lower starch concentrations lead to slower, less controlled growth, producing irregular quasi-spherical geometries.^2,40^

**Fig. 13:**
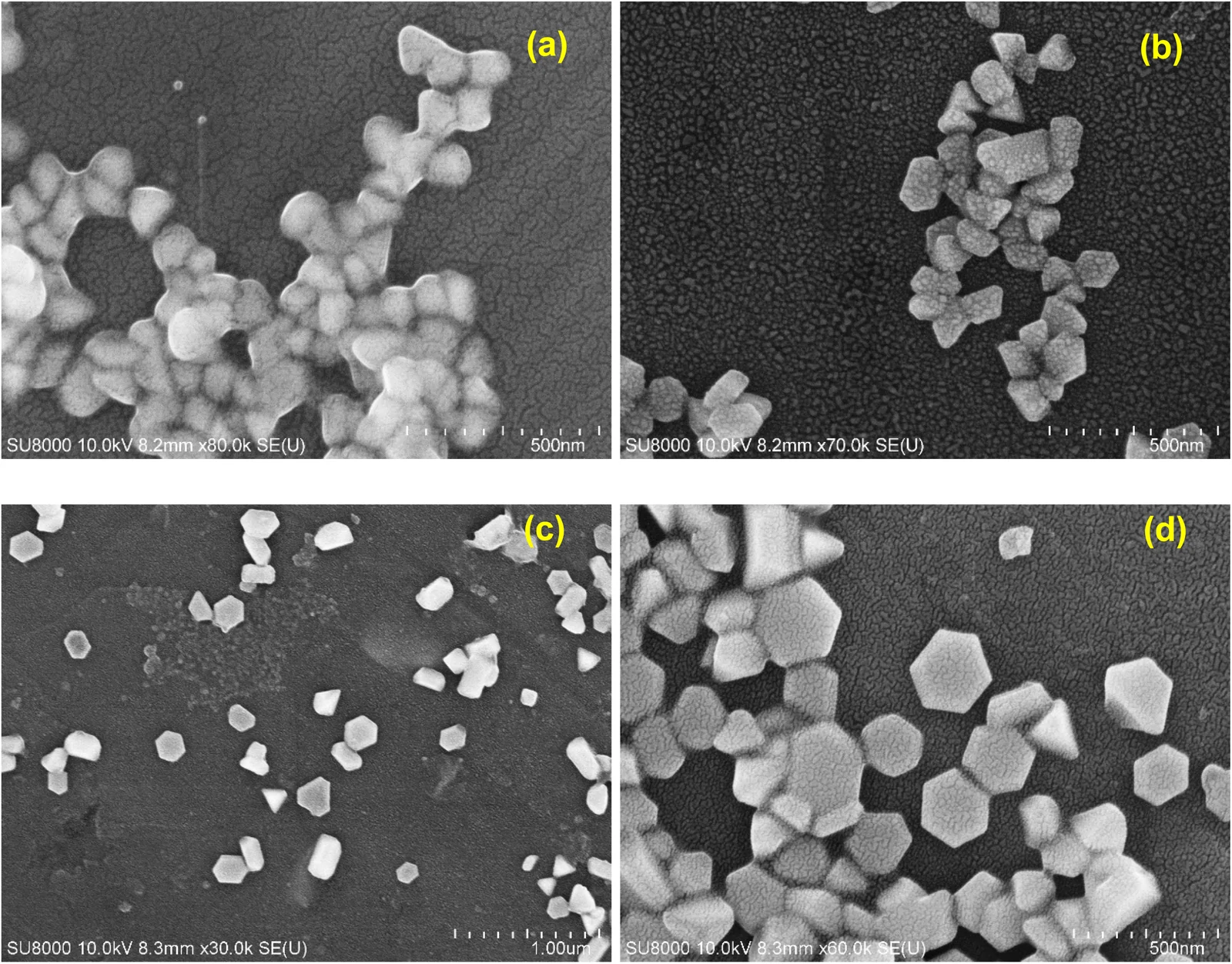
SEM images of the bismuth nanoplates synthesized in the presence of PVP using different amounts of starch as a reductant (a) 1.0 g, (b) 1.5 g, (c) 2.0 g, and (d) 2.5 g (conditions: PVP = 1.0 g, time = 2 h, temperature = 100 °C, pH = 13).

Because the optical response of these anisotropic Bi nanoplates can be tuned across the visible-to-near-infrared regime (410 to 600 nm) by varying synthetic parameters, these nanostructures possess optical properties that make them promising candidates for future investigations in photothermal conversion, optical sensing, and thermoelectrics.

## Conclusion

In summary, phase-pure rhombohedral bismuth (Bi^0^) nanoplates were successfully synthesized *via* a facile, bio-assisted aqueous reduction route using starch as a kinetic regulator and PVP as a facet-selective capping agent. Powder XRD confirms high crystal purity (JCPDS Card No. 44-1246), while FTIR, TGA, and zeta potential (−50 mV) analyses demonstrate a robust dual-polymer capping layer anchored by starch hydroxyl hydrogen bonding (redshifted to 3290 cm^−1^) and weak physical adsorption of PVP (1655 cm^−1^ C=O stretch). Quantitative statistical SEM analysis (*n* > 30) reveals that lower starch concentrations produce quasi-spherical nanoparticles (∼80 nm), whereas higher starch levels accelerate reduction kinetics to favor uniform hexagonal nanoplates (145–240 nm). Concurrently, increasing the PVP feed ensures effective passivation of high-energy crystal faces, transitioning the morphology from mixed irregular nanoplates (∼115 nm) to predominant hexagonal nanoplates (120–145 nm). Time-resolved observations show an initial stage at 2 h consisting of a mixed morphology (∼145 nm) that evolves beyond 2 h into uniform hexagonal nanoplates (∼200 nm), ultimately developing a visibly increased thickness profile at 24 h *via* Ostwald ripening coupled with kinetic facet capping. This 2D structural anisotropy drives a pronounced bathochromic shift in the localized surface plasmon resonance (LSPR) from 410 nm to ∼600 nm, originating from in-plane dipolar plasmon modes. Overall, this study establishes a reproducible green synthesis strategy for precisely tailoring the dimensional and plasmonic features of bismuth nanostructures, laying a solid foundation for their integration into sensing, photothermal, thermoelectric, and energy-conversion technologies.

## Author contributions

M.M. Conducted the experimental works, formal analysis, and data curation; P.S. conducted some instrumental analysis and data curation; B.O supervised the research study; M.M. & P.S wrote the manuscript; M.M. & B.O. review and edit the manuscript. All authors gave final approval for publication and agreed to be held accountable for the work performed therein.

## Conflicts of interest

The authors declare no competing interest.

## Data Availability

All data generated or analyzed during this study are included in this article. Backup data are available from the corresponding author(s) upon reasonable request. While preparing this work, the author(s) used “*Gemini Advance*” to paraphrase and edit the language. After using those tools, the author(s) reviewed and revised the content as needed and take(s) full responsibility for the publication’s content.
